# Supplementary material for: PIKfyve, expressed by CD11c-positive cells, controls tumor immunity
Source: Nat Commun. 2024 Jun 28;15:5487. doi: 10.1038/s41467-024-48931-9 (PMC11213953; doi:10.1038/s41467-024-48931-9)
Supplement: Supplementary file 1 — Supplementary Information [file 41467_2024_48931_MOESM1_ESM.pdf]

PIKfyve, expressed by CD11c-positive cells, controls tumor immunity

Jae Eun Choi[1,2,11], Yuanyuan Qiao[1,2,3], Ilona Kryczek[4,5], Jiali Yu[4,5], Jonathan Gurkan[1,2, 12], Yi Bao[1,2], Mahnoor Gondal[1,2,6], Jean Ching-Yi Tien[1,2], Tomasz Maj[4,5], Sahr Yazdani[1,2, 13], Abhijit Parolia[1,2], Houjun Xia[4,5], JiaJia Zhou[4,5], Shuang Wei[4,5], Sara Grove[4,5], Linda Vatan[4,5], Heng Lin[4,5], Gaopeng Li[4,5], Yang Zheng[1,2], Yuping Zhang[1,2], Xuhong Cao[1,2,7], Fengyun Su[1,2], Rui Wang[1,2], Tongchen He[1,2], Marcin Cieslik[1,2,6], Michael D. Green[5,8,9], Weiping Zou[1,2,3,4,5 *], and Arul M. Chinnaiyan[1,2,3,7,10*]

[1]Department of Pathology, University of Michigan, Ann Arbor, MI, USA
[2]Michigan Center for Translational Pathology, University of Michigan, Ann Arbor, MI, USA
[3]Rogel Cancer Center, University of Michigan, Ann Arbor, MI, USA
[4]Department of Surgery, University of Michigan, Ann Arbor, MI, USA
[5]Center of Excellence for Cancer Immunology and Immunotherapy, University of Michigan, Ann Arbor, MI, USA
[6]Department of Computational Medicine & Bioinformatics, University of Michigan, Ann Arbor, MI, USA
[7]Howard Hughes Medical Institute, University of Michigan, Ann Arbor, MI, USA
[8]Department of Radiation Oncology, University of Michigan, Ann Arbor, MI, USA
[9]Department of Radiation Oncology Veterans Affairs Ann Arbor Healthcare System, Ann Arbor, MI, USA
[10]Department of Urology, University of Michigan, Ann Arbor, MI, USA
[11]Present address: Department of Pediatrics, University of California, San Francisco, CA, USA
[12]Present address: Feinberg School of Medicine, Northwestern University, Chicago, IL, USA
[13]Present address: Department of Pediatrics, Children's Hospital of Philadelphia, PA, USA

*Jointly corresponding authors:

Arul M. Chinnaiyan, M.D., Ph.D.
Email: arul@umich.edu

Weiping Zou, M.D., Ph.D.
Email: wzou@umich.edu

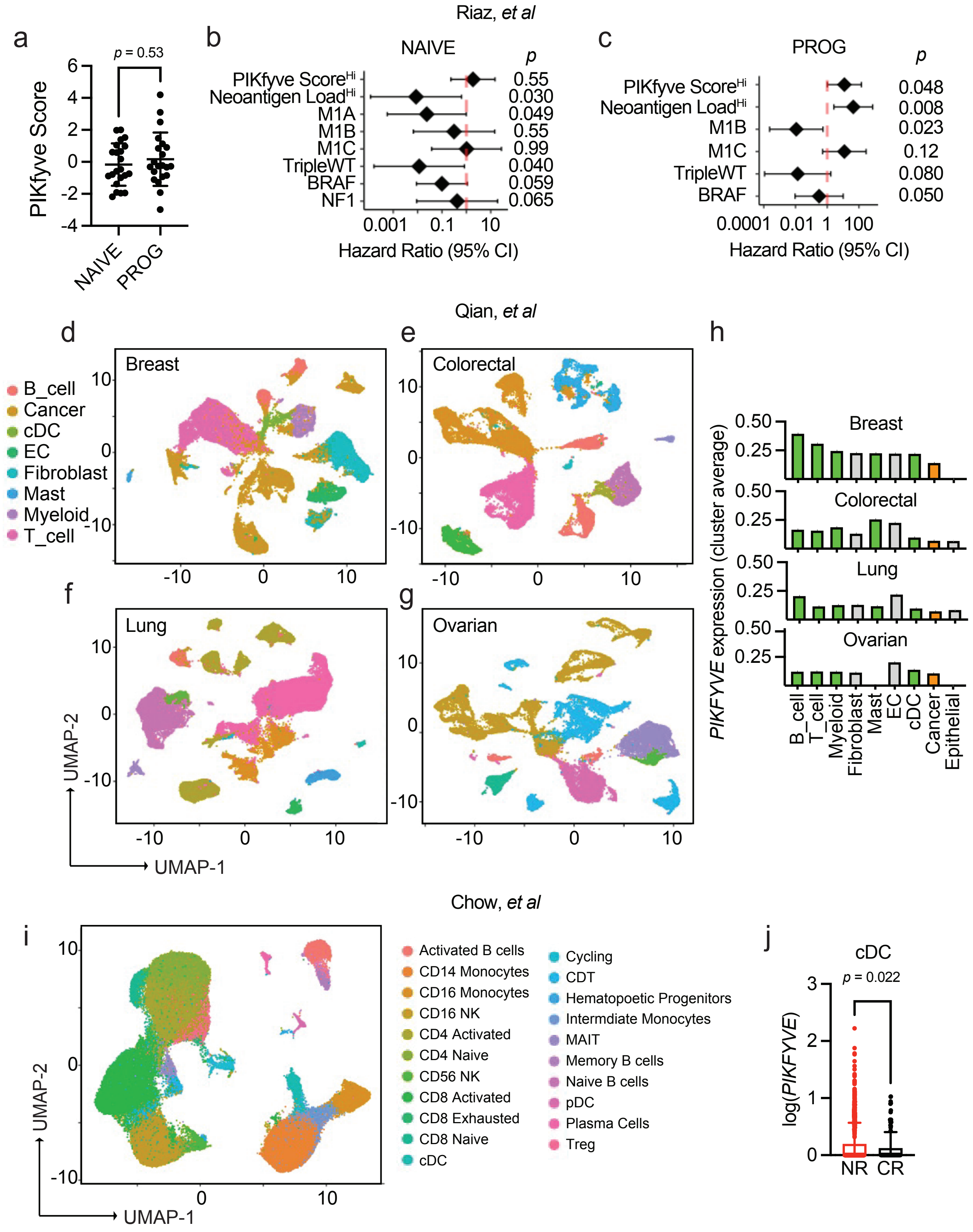

Riaz, et al
a
p = 0.53
PIKfyve Score
NAIVE
PROG
b
NAIVE
p
PIKfyve Score^Hi 0.55
Neoantigen Load^Hi 0.030
M1A 0.049
M1B 0.55
M1C 0.99
TripleWT 0.040
BRAF 0.059
NF1 0.065
Hazard Ratio (95% CI)
c
PROG
p
PIKfyve Score^Hi 0.048
Neoantigen Load^Hi 0.008
M1B 0.023
M1C 0.12
TripleWT 0.080
BRAF 0.050
Hazard Ratio (95% CI)
Qian, et al
d
Breast
B_cell
Cancer
cDC
EC
Fibroblast
Mast
Myeloid
T_cell
e
Colorectal
f
Lung
g
Ovarian
UMAP-2
UMAP-1
h
Breast
Colorectal
Lung
Ovarian
PIKFYVE expression (cluster average)
B_cell
T_cell
Myeloid
Fibroblast
Mast
EC
cDC
Cancer
Epithelial
Chow, et al
i
UMAP-2
UMAP-1
Activated B cells
CD14 Monocytes
CD16 Monocytes
CD16 NK
CD4 Activated
CD4 Naive
CD56 NK
CD8 Activated
CD8 Exhausted
CD8 Naive
cDC
Cycling
CDT
Hematopoetic Progenitors
Intermdiate Monocytes
MAIT
Memory B cells
Naive B cells
pDC
Plasma Cells
Treg
j
cDC
p = 0.022
log(PIKFYVE)
NR
CR

Supplementary Information 1. Characterization of *PIKFYVE* and PIKfyve score expression in cancer patient tumors. a) Comparison of PIKfyve scores in patients with melanoma who were ICB treatment-naïve ("NAÏVE") versus those who had previously progressed on ICB treatment ("PROG"). Data plotted are mean ± s.d. from bulk RNA-seq data.[44] *P* value is determined by Mann Whitney U test. **b)** Forest plot of hazard ratios of NAÏVE cohort by high versus low PIKfyve score, high versus low neoantigen load, disease stage (reference is M0), and mutation subtype (reference is None). Data plotted are hazard ratios with 95% confidence intervals from bulk RNA-seq data.[44] *P* values are determined by a multivariate cox proportional hazards model. **c)** Forest plot of hazard ratios of PROG cohort by high versus low PIKfyve score, high versus low neoantigen load, disease stage (reference is M1A), and mutation subtype (reference is None). Data plotted are hazard ratios with 95% confidence intervals from bulk RNA-seq data.[44] *P* values are determined by a multivariate cox proportional hazards model. T-SNE plots of immune cells in pre-treatment tumors from patients with **(d)** breast, **(e)** colorectal, **(f)** lung or **(g)** ovarian cancer from scRNA-seq data.[48] **h)** Barplots of *PIKFYVE* cluster average expression by cancer, stroma, or immune cell type from scRNA-seq data.[48] **i)** T-SNE plot of immune cells in pre-treatment tumors from patients with endometrial cancer from scRNA-seq data.[50] **j)** Comparison of log (*PIKFYVE*) expression in individual cDCs in patients with endometrial cancer who were nonresponders ("NR" including SD and PD) versus CR to ICB treatment. Data plotted are mean ± s.d. of all individual cDCs from scRNA-seq data.[50] *P* value is determined by student t-test with Welch's correction. All *P* values are two-sided. Source data are provided in Supplementary Data 9.

a

Gating strategy for Supp 2b-g

Subset	Gating Strategy
Naïve	$CD3^{+}CD8^{+}CD44^{Lo}CD62L^{+}$
Terminally differentiated	$CD3^{+}CD8^{+}CD44^{Hi}$KLRG1+
Resident Memory	$CD3^{+}CD8^{+}CD44^{Hi}KLRG1^{-}$CD49a+
Exhausted	$CD3^{+}CD8^{+}CD44^{Hi}$KLRG1-CD49a-$TIM3^{+}PD\text{-}1^{+}$
Central memory	$CD3^{+}CD8^{+}CD44^{Hi}$KLRG1-CD49a-$TIM3^{-}PD\text{-}1^{-}CD62L^{+}$
$CD69^{+}$ Effector	$CD3^{+}CD8^{+}CD44^{Hi}$KLRG1-CD49a-$TIM3^{-}PD\text{-}1^{-}CD62L^{-}CD69^{+}$
$CD69^{-}$ Effector	$CD3^{+}CD8^{+}CD44^{Hi}$KLRG1-CD49a-$TIM3^{-}PD\text{-}1^{-}CD62L^{-}CD69^{-}$

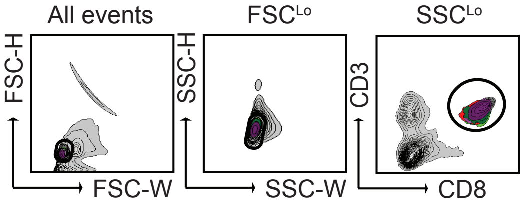

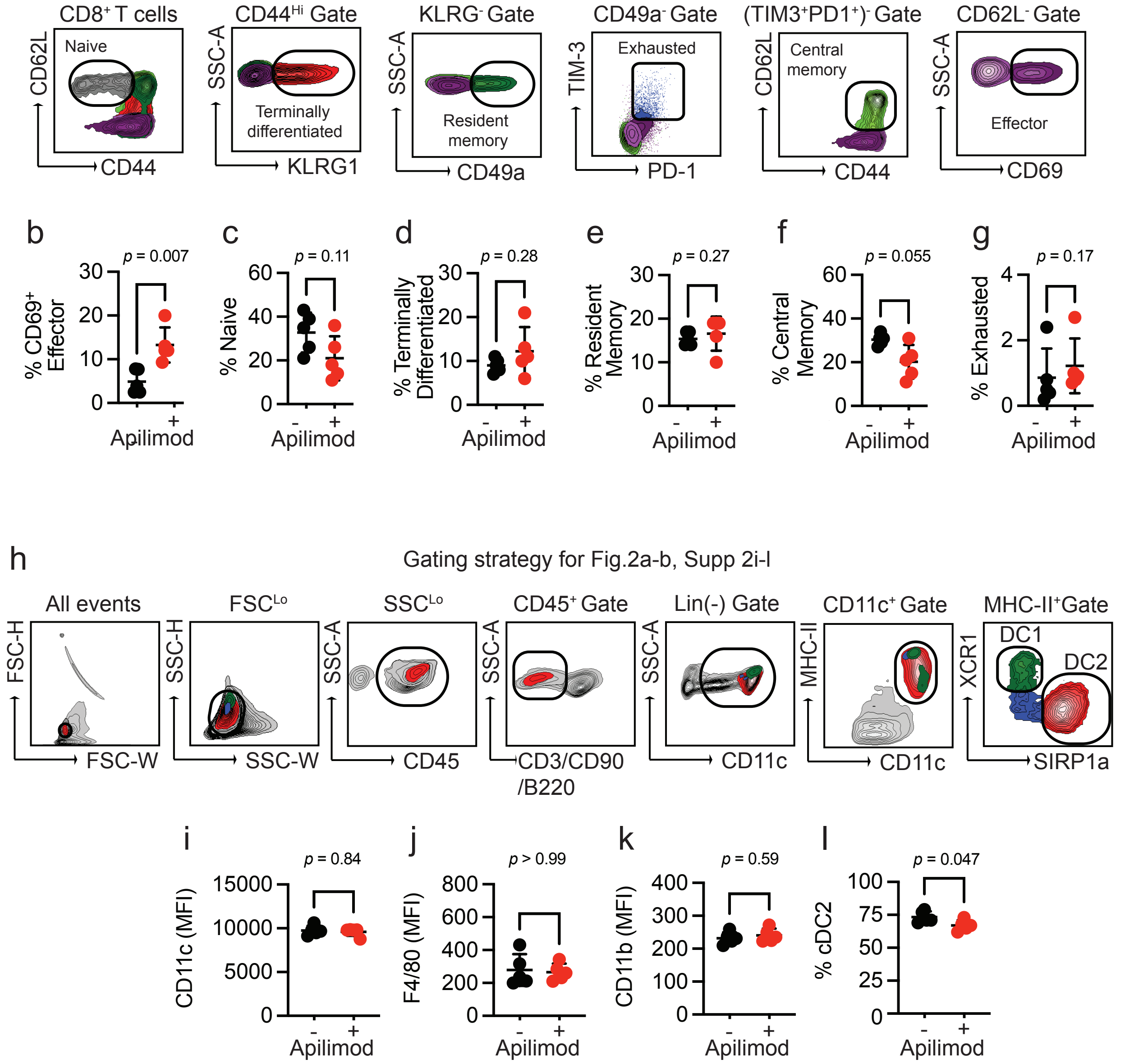

Supplementary Information 2: Characterization of T cell and myeloid subsets of PIKfyve inhibitor-treated splenic immune cells. a) Gating strategy for Supp. 2 panels b-g for $CD8^{+}$ T cells. Comparison of the percentage of **(b)** $CD69^{+}$ effector, **(c)** naïve, **(d)**

terminally differentiated, **(e)** resident memory, **(f)** central memory, and **(g)** exhausted $CD8^+$ T cells from spleens of non-tumor-bearing mice treated with vehicle or apilimod (30 mg/kg daily) on day 5 of treatment (n = 5 spleens per group). Data plotted are mean ± s.d. *P* values are determined from student t-test. **h)** Gating strategy for Figure 2 panels a-b and Supp. 2 panels i-l for myeloid cells and cDCs. Comparison of the percentage of total **(i)** CD11c, **(j)** F4/80, and **(k)** CD11b in $CD45^+$ cells and (l) percentage of cDC2 cells from spleens of non-tumor-bearing mice treated with vehicle or apilimod (30 mg/kg daily) on day 5 of treatment (n = 5 per group). Data plotted are mean ± s.d. *P* values are determined from student t-test. All *P* values are two-sided without corrections for multiple comparisons. Source data are provided in Supplementary Data 9.

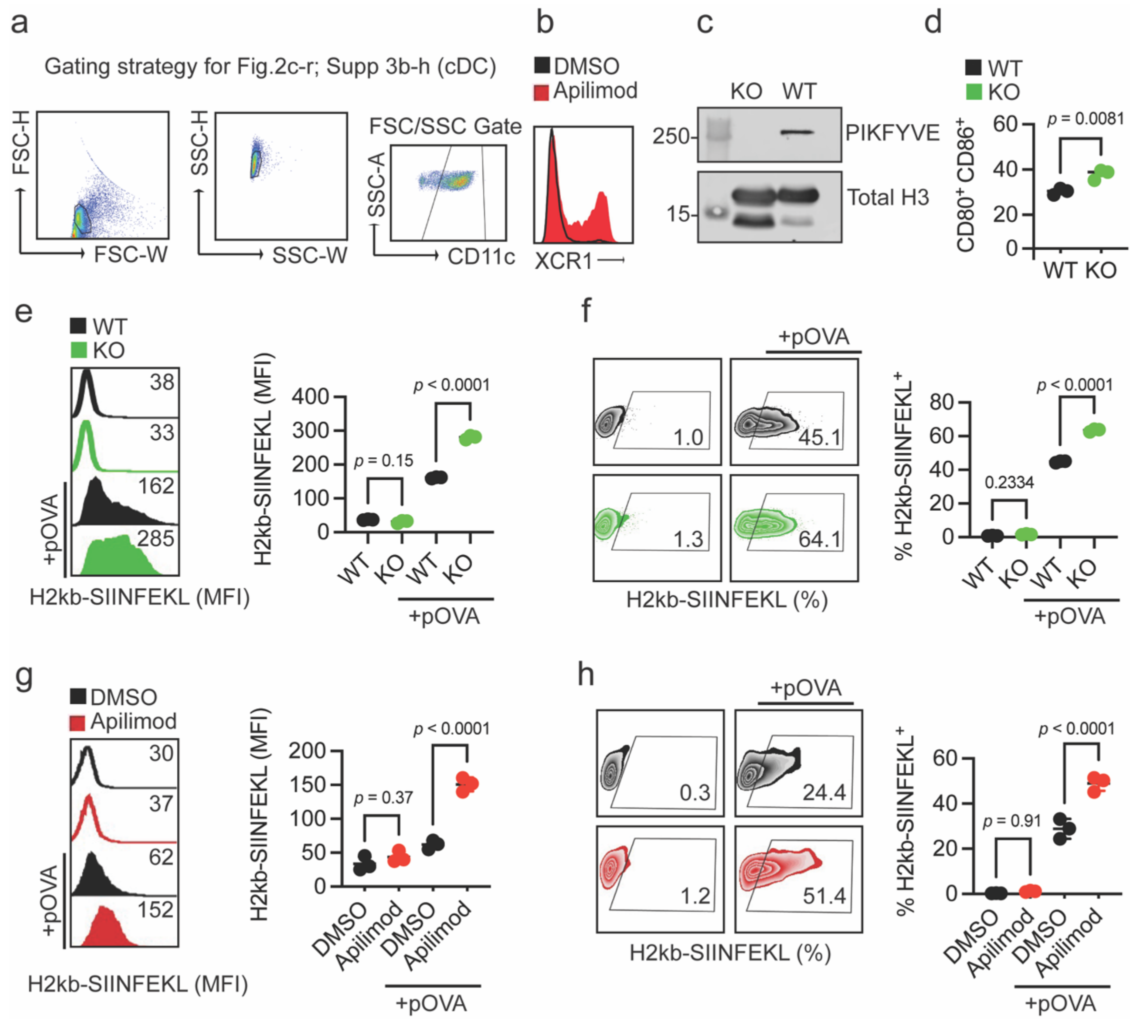

Supplementary Information 3: PIKfyve expression CD11c^{+} cells and antigen presentation. a) Gating strategy for Figure 2 panels c-r and Supp. panels b-h (CD11c^{+} gate). **b)** Representative density plot of median fluorescent intensity of surface XCR1 in DMSO or apilimod-treated cDCs after 20 hours on culture day 6. **c)** Immunoblots of PIKfyve in *Pikfyve* WT vs. KO cDC lysates on culture day 9. Total histone H3 serves as loading control. Image is representative of two experiments. **d)** Comparison of the percentage of CD80^{+}CD86^{+-} cDCs in *Pikfyve* WT vs KO models. Data plotted are mean ± s.d. *P* values are determined from student t-tests across 3 biological replicates. Relative median fluorescent intensity of surface H-2kb-SIINFEKL **(e)** and percent H-2kb-SIINFEKL^{+} cells **(f)** in *Pikfyve* WT vs. KO cDC +/- pOVA (100 ng/ml) after 12 hours on culture day 9. Data plotted are mean ± s.d. *P* value is determined by student t-test across 3 biological replicates. Representative plots are shown. Relative median fluorescent intensity of surface H-2kb-SIINFEKL **(g)** and percent H2kb-SIINFEKL^{+} cells **(h)** in DMSO or apilimod-treated cDCs +/- pOVA (100 ng/ml) after 12 hours. Data plotted are mean ± s.d. *P* value is determined by student t-test across 3 biological replicates. Representative plots are shown. All *P* values are two-sided. Source data are provided in Supplementary Data 9. Source data for immunoblot in Supp 3c provided in Supplementary Information 11.

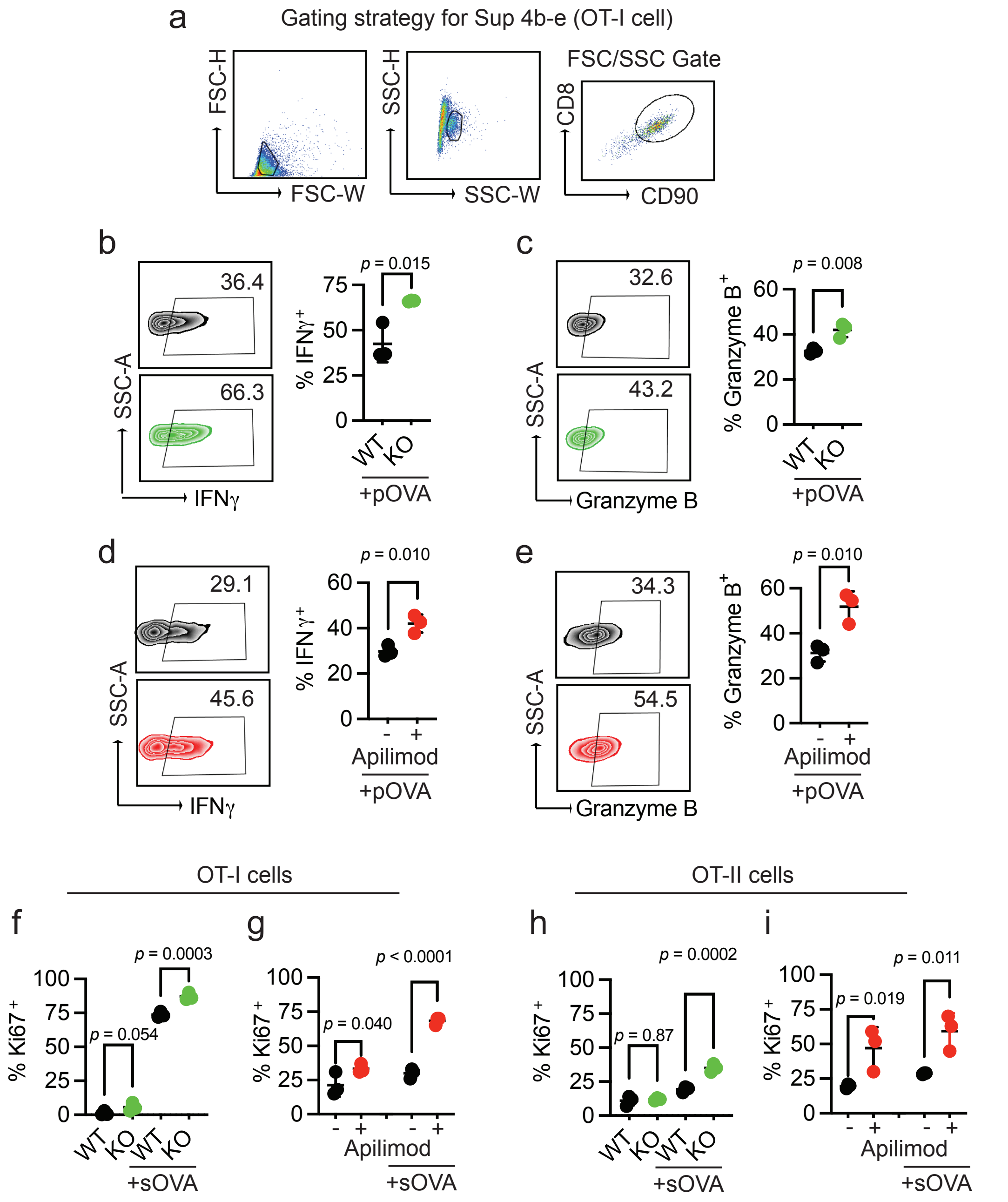

Supplementary Information 4. PIKfyve expression in CD11c⁺ cells and OT-I and OT-II T cell activation. a) Gating strategy for Supp. 4b-e (CD90$^+$ CD8$^+$ gate for OT-I cells). Representative dot plots and percentages of **(b)** IFNγ$^+$ and **(c)** granzyme B$^+$ OT-I cells after 48 hours of co-culture with *Pikfyve* WT vs. KO cDCs +/- pOVA 100ng/ml. *n* = 3 biological replicates. Data plotted are mean ± s.d. *P* value is determined by student t-test. Representative dot plot and percentage of **(d)** IFNγ$^+$ and **(e)** granzyme B$^+$ OT-I cells after 48 hours of co-culture with DMSO or apilimod pre-treated cDCs +/- pOVA 100 ng/ml. *n* = 3 biological replicates. Data plotted are mean ± s.d. *P* value is determined by student t-test. Percentage of Ki67$^+$ OT-I cells after 72 hours of co-culture with **(f)** *Pikfyve*

WT versus KO pre-treated cDCs +/- sOVA (10 µg/ml) or **(g)** DMSO or apilimod pre-treated cDCs +/- sOVA (10 µg/ml). Percentage of Ki67+ OT-II cells after 72 hours of co-culture with **(h)** *Pikfyve* WT versus KO pre-treated cDCs +/- sOVA or **(i)** DMSO or apilimod pre-treated cDCs +/- sOVA. n = 3 biological replicates. Data plotted are mean ± s.d. *P* value is determined by student t-test. All *P* values are two-sided. Source data are provided in Supplementary Data 9.

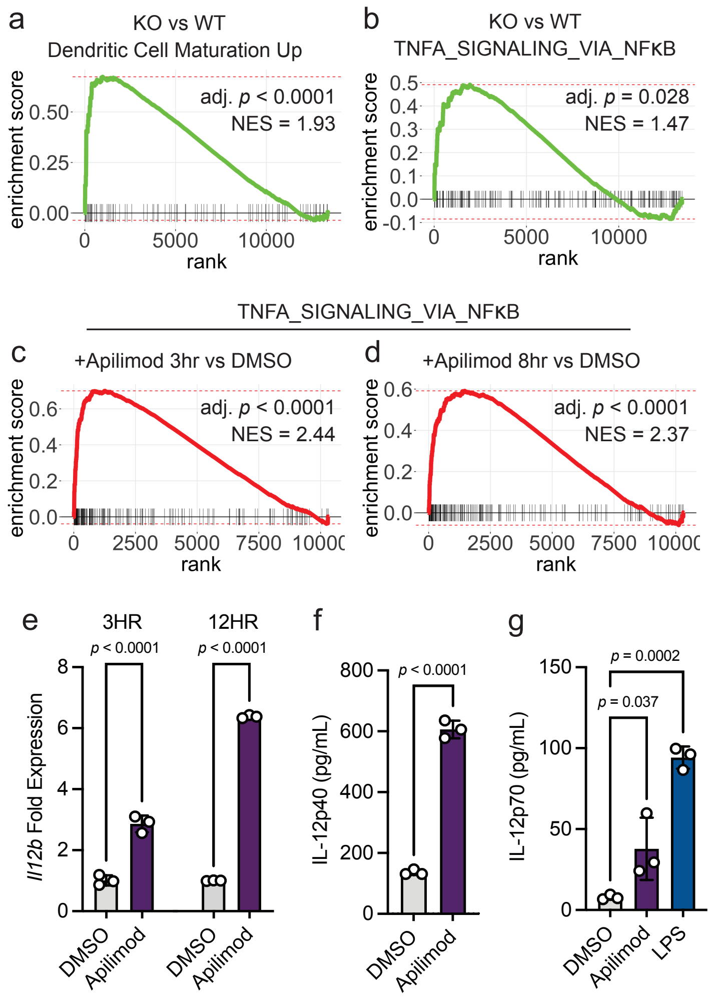

Supplementary Information 5: PIKfyve expression in CD11c^{+} cells and dendritic cell gene signatures and Il-12 expression. a) Enrichment plot of Dendritic Cell Maturation Up gene signature (M4562: LENAOUR_DENDRITIC_CELL_MATURATION_UP) in *Pikfyve* KO versus WT cDCs on culture day 9. **b)** Enrichment plot of MSigDB Hallmark "TNF_SIGNALING_VIA_NFκB" in *Pikfyve* KO versus WT cDCs on culture day 9. Enrichment plots of MSigDB Hallmark "TNF_SIGNALING_VIA_NFκB" in apilimod versus DMSO-treated cDCs treated for **(c)** 3 hours or **(d)** 8 hours on culture day 6. **e)** Comparison of *Il12b* expression fold change by RT-qPCR in apilimod vs. DMSO-treated cDCs treated for 3 or 12 hours on culture day 6. Data plotted are mean ± s.d. *P* value is determined by ANOVA after post-hoc Tukey adjustment for multiple comparisons across 3 biological replicates**. f)** Comparison of IL-12p40 protein levels by ELISA in media from apilimod versus DMSO-treated cDCs treated for 12 hours on culture day 6. Data plotted are mean ± s.d. *P* values are generated from student t-test across 3 biological replicates. **g)** Comparison of IL-12p70 protein levels by ELISA in media from apilimod vs. DMSO-treated cDCs treated for 24 hours on culture day 6. Data plotted are mean ± s.d. *P* values are generated from student t-test across 3 biological replicates. All *P* values are two-sided. Source data are provided in Supplementary Data 9.

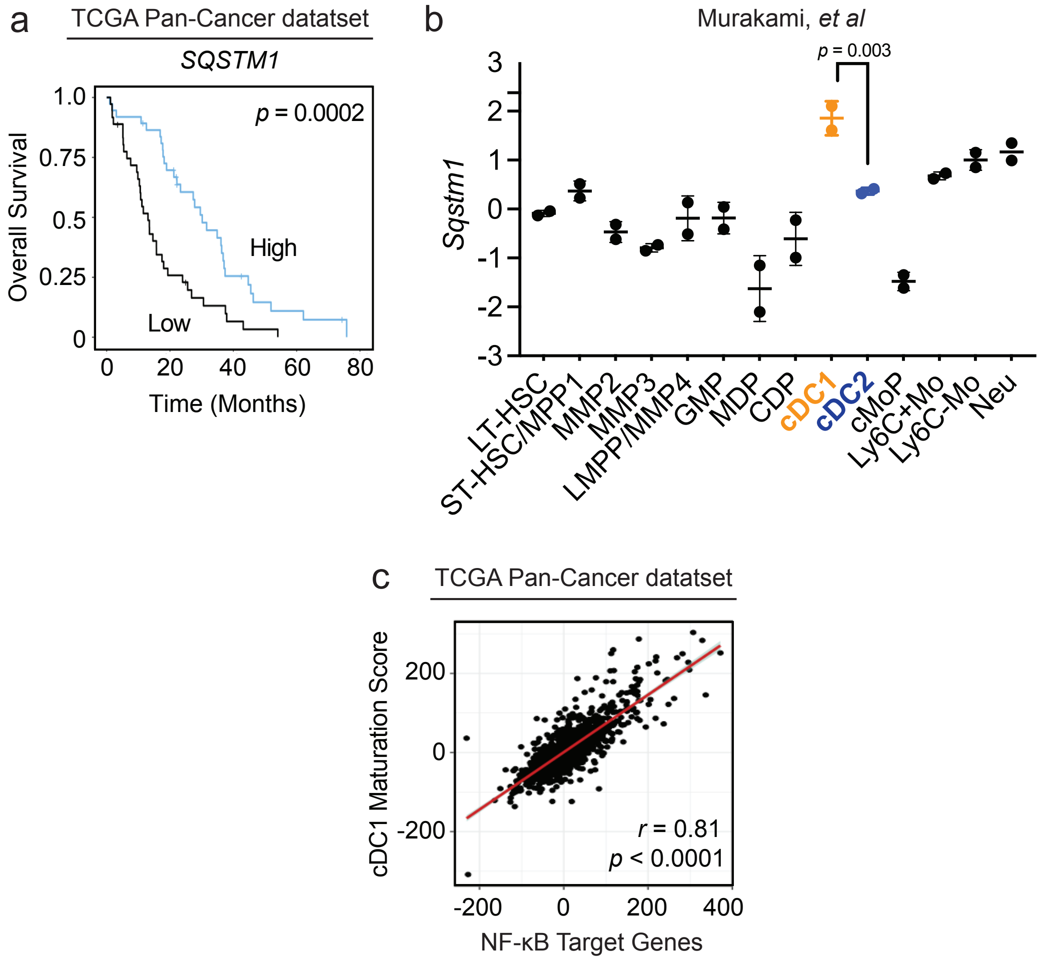

Supplementary Information 6. Comparison of NF-κB target genes in cDC subsets. a) Kaplan-Meier curve of overall survival of patients in the TCGA Pan-Cancer bulk RNA-seq dataset, by high or low (median) *SQSTM1* normalized gene expression. *P* value is determined by log-rank test. **b)** Comparison of *Sqstm1* normalized gene expression across myeloid lineage cell types. Data plotted are mean ± s.d. from bulk RNA-seq data.[81] *P* value is determined by ANOVA after post-hoc comparisons across all pair-wise comparisons. *P* value for cDC1 versus cDC2 shown on plot. **c)** Correlation between cDC1 maturation score and NF-κB target genes expression in the TCGA Pan-Cancer bulk RNA-seq dataset. Correlation coefficient and *P* value are calculated using Pearson's product-moment correlation. All *P* values are two-sided. Source data are provided in Supplementary Data 9.

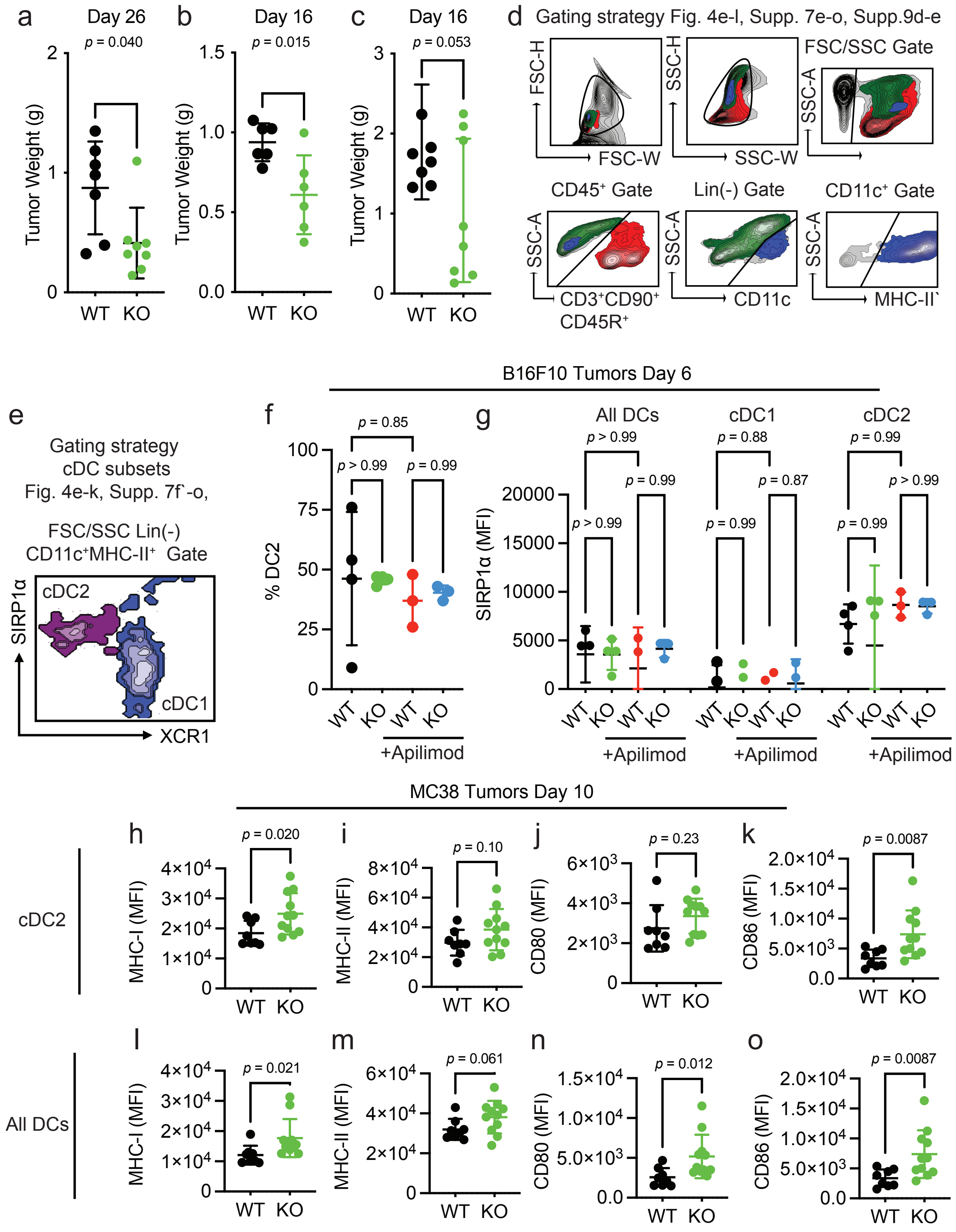

Supplementary Information 7. Comparison of tumor-associated cDC subsets with genetic loss of *Pikfyve* in CD11c⁺ cells. Tumor weights for **(a)** MC38 tumor measured

on Day 26, **(b)** MCA-205 tumor measured on Day 16, and **(c)** B16F10 tumor measured on Day 16. Data plotted are mean ± s.d. *P* values are determined by Mann-Whitney U test. **d)** Gating strategy for Fig. 4e-l, Supp. 7e-o, Supp. 9d-e. $CD45^+$ Lin(-) $CD11c^+$ MHC-II^+ gate for dendritic cells from tumors and tumor-draining lymph nodes (TDLN). **e)** Gating strategy for Fig. 4e-k, Supp. 7f-o. Additional cDC1 and cDC2 gates for dendritic cells from tumors. B16F10 tumor model. **(f)** % cDC2 of all DC cells and **(g)** SIRP1α median fluorescent intensity by all DC, cDC1 or cDC2 subset in *Pikfyve* KO mice or WT mice treated with vehicle or apilimod (30 mg/kg daily). Bilateral subcutaneous tumors from each mouse (*n* = 4 per group) were combined for analysis. Data plotted are mean ± s.d. *P* value is determined by ANOVA after post-hoc Tukey adjustment for multiple comparisons on day 6 of treatment. **(h)** MHC-I, **(i)** MHC-II, **(j)** CD80 and **(k)** CD86 median fluorescent intensity in the cDC2 subset and **(l)** MHC-I, **(m)** MHC-II, **(n)** CD80 and **(o)** CD86 median fluorescent intensity in all DCs in MC38 tumors of *Pikfyve* KO (*n* = 11) or WT mice (*n* = 8). Data plotted are mean ± s.d. *P* value is determined by student t-test with Welch's correction on day 10. All *P* values are two-sided. Source data are provided in Supplementary Data 9.

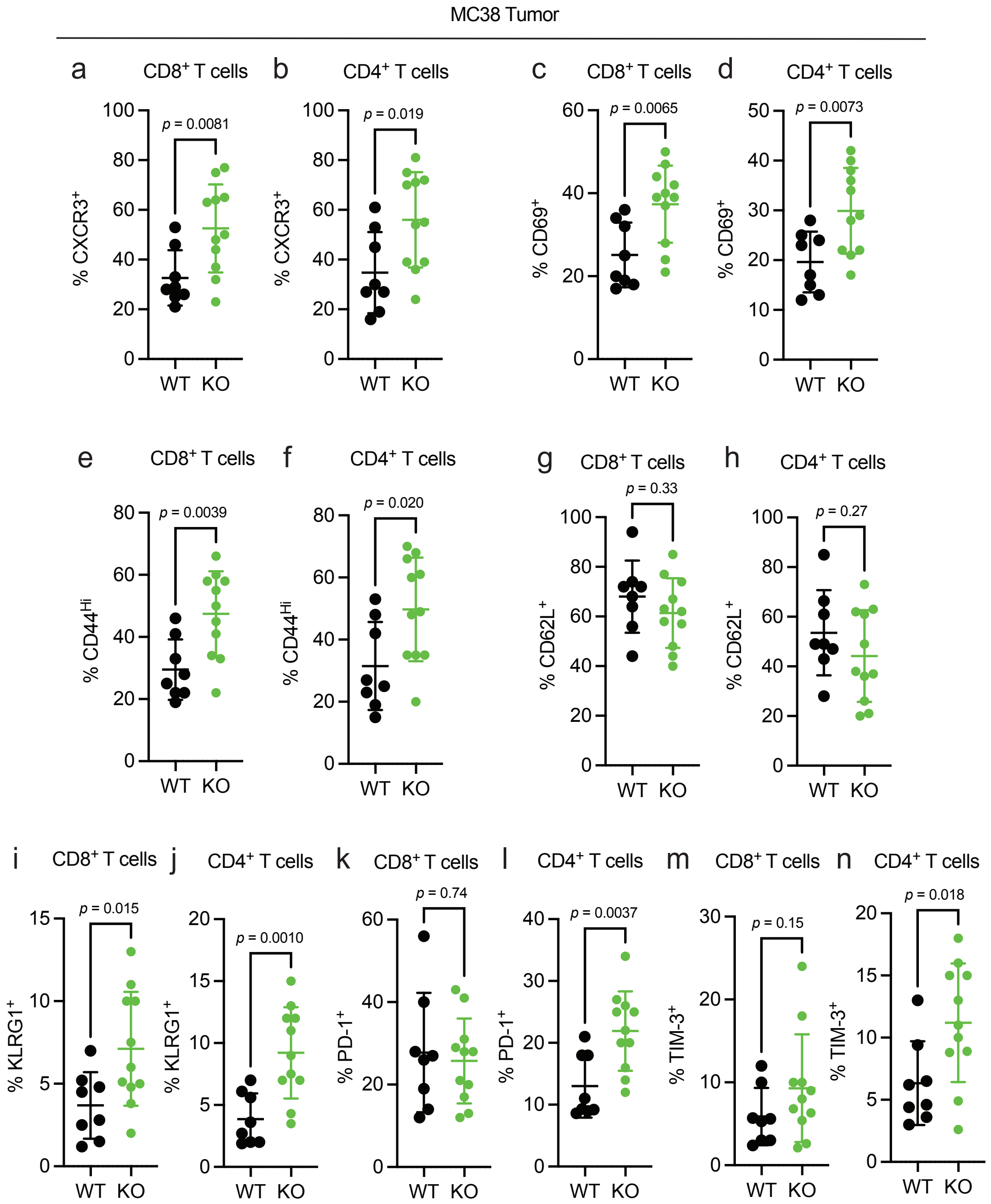

Supplementary Information 8. Comparison of tumor-associated T cell subsets by expression of *Pikfyve* in CD11c$^+$ cells. Comparison of the percentage of (**a**) CXCR3$^+$, (**c**) CD69$^+$, (**e**) CD44^{Hi}, (**g**) CD62L$^+$, (**i**) KLRG1$^+$, (**k**) PD-1$^+$ and (**m**) TIM-3$^+$ CD8$^+$ T cells and (**b**) CXCR3$^+$, (**d**) CD69$^+$, (**f**) CD44^{Hi}, (**h**) CD62L$^+$, (**j**) KLRG1$^+$, (**l**) PD-1$^+$ and (**n**) TIM-3$^+$ CD4$^+$ T cells. T cells isolated from MC38 tumors of *Pikfyve* KO (n = 11) or WT mice (n = 8). Data plotted are mean ± s.d. P value is determined by student t-test with Welch's correction on day 10. All P values are two-sided without corrections for multiple comparisons. Source data are provided in Supplementary Data 9.

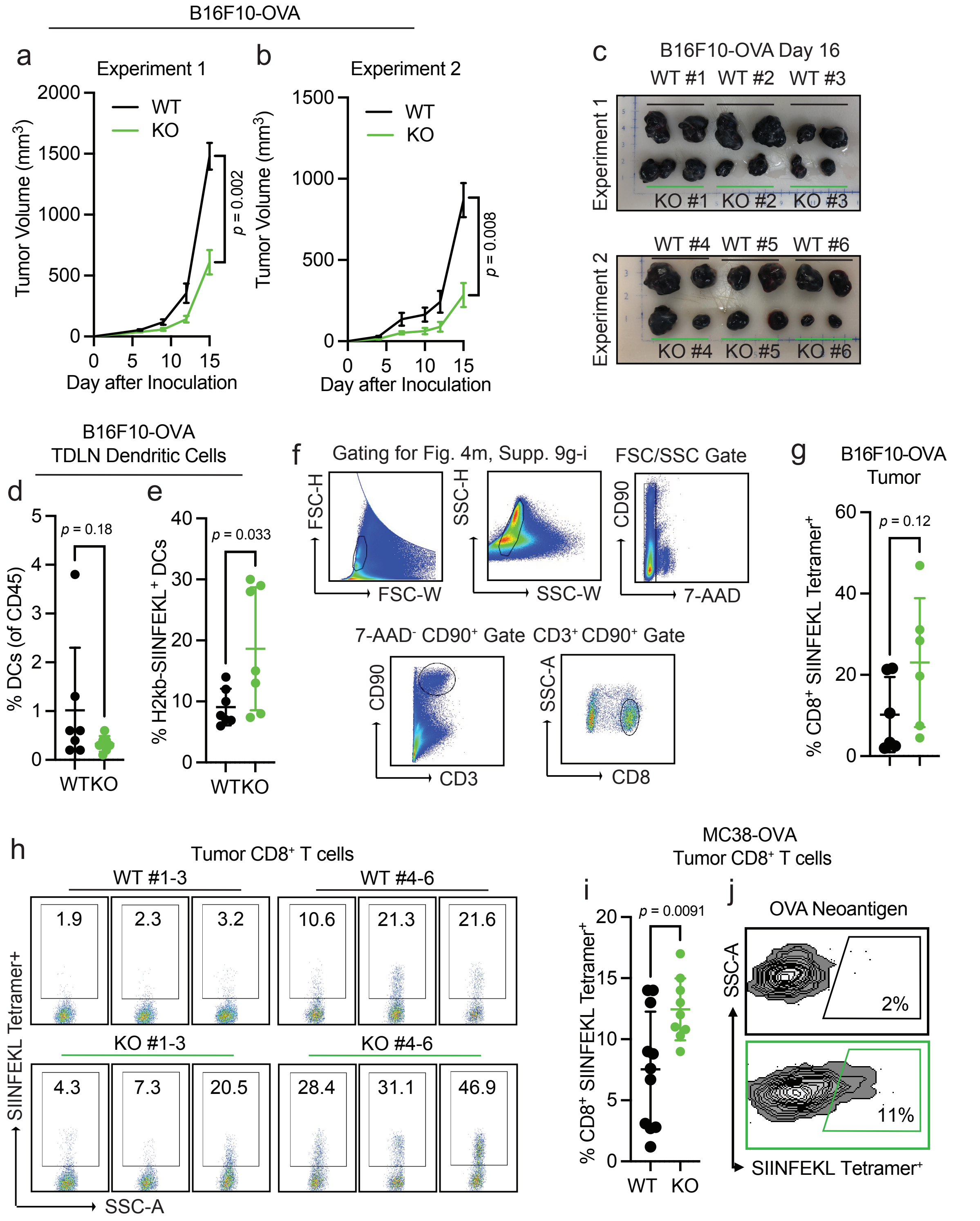

Supplementary Information 9: Characterization of tumor-associated T cell responses with genetic loss of *Pikfyve* in CD11c^{+} cells. Subcutaneous tumor **(a)** Experiment 1 and **(b)** Experiment 2 of B16F10-OVA tumors (mm^{3}) in *Pikfyve* KO mice or WT mice. Data plotted are mean ± s.e.m. *P* value is determined by Mann-Whitney U

test on Day 15. **(c)** Image of B16F10-OVA tumors on Day 16. Bilateral subcutaneous tumors were excised from 3 WT/KO littermate pairs per experiment in two independent experiments. **d)** Percent dendritic cells of $CD45^+$ cells in *Pikfyve* WT (*n* = 6) versus KO (*n* = 6) tumor-draining lymph nodes of B16F10-OVA tumors. Data plotted are mean ± s.d. *P* value is determined by Mann-Whitney U test. **e)** Percent H2-kb-$SIINFEKL^+$ dendritic cells in WT (*n* = 6) versus KO (*n* = 6) tumor-draining lymph nodes of B16F10-OVA tumors. Data plotted are mean ± s.d. *P* value is determined by Mann-Whitney U test. **f)** Gating strategy for Fig. 4m, Supp. 9g-i (7-AAD^- $CD90^+$ $CD3^+$ $CD8^+$ gate for intratumoral CD8+ T cells). **(g)** Percentage and **(h)** dot plots of SIINFEKL $Tetramer^+$ $CD8^+$ T cells isolated from B16F10-OVA tumors. Bilateral subcutaneous tumors from each WT (*n* = 6) or KO (*n* = 6) mouse were combined for analysis. WT/KO littermate pairs #1-3 (Experiment 1) and pairs #4-6 (Experiment 2) are from two independent experiments. Data plotted are mean ± s.d. *P* value is determined by student t-test with Welch's correction. **(i)** Percentage and **(j)** dot plots of SIINFEKL $Tetramer^+$ $CD8^+$ T cells isolated from MC38-OVA WT (*n* = 11) or KO (*n* = 9) tumors. Data plotted are mean ± s.d. *P* value is determined by student t-test with Welch's correction. All *P* values are two-sided. Source data are provided in Supplementary Data 9.

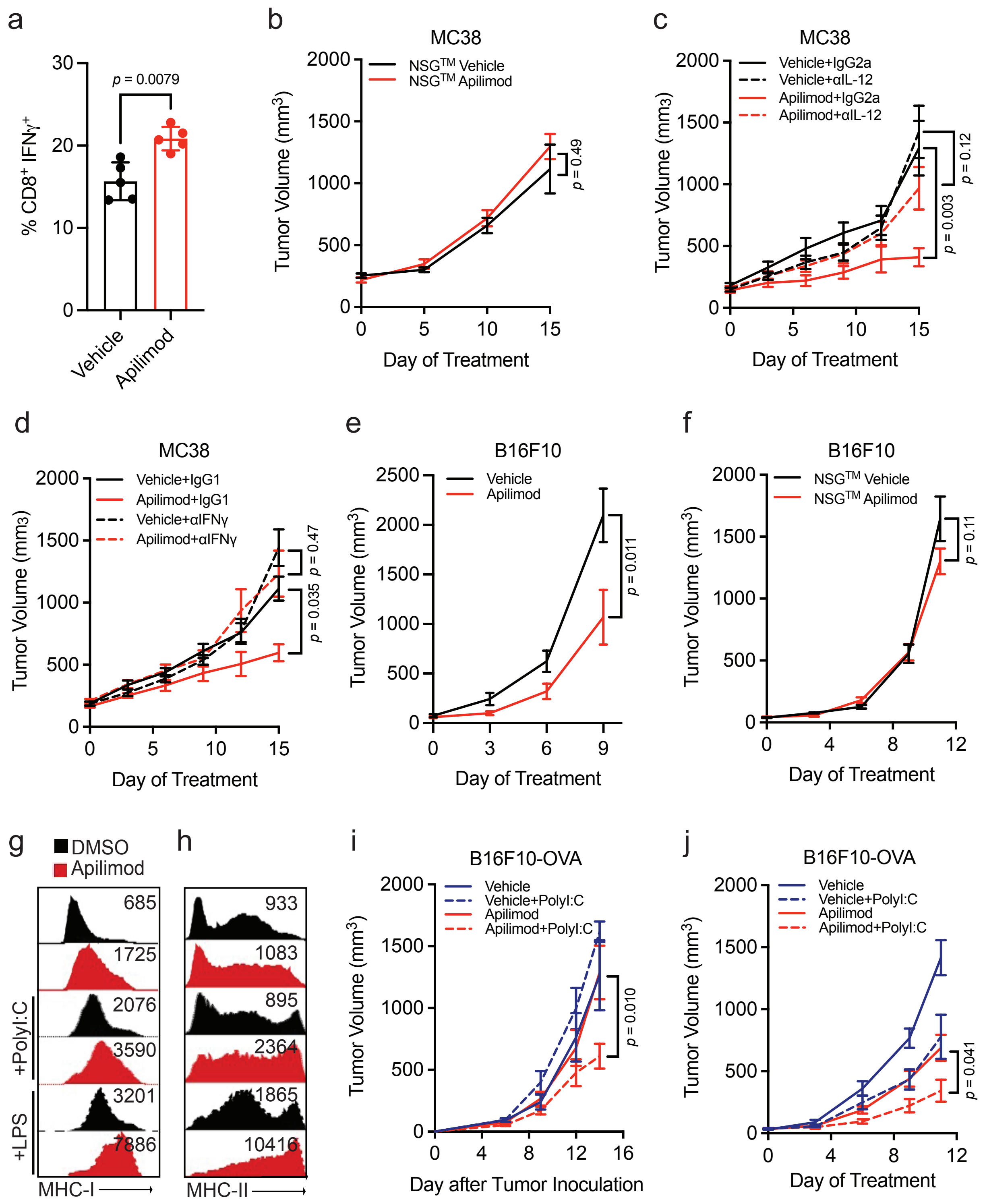

Supplementary Information 10: PIKfyve inhibitor anti-tumor efficacy with loss of immune signaling pathways. a) Percent IFNγ^+ of CD8$^+$ T cells in vehicle or apilimod-treated MC38 tumors. Bilateral subcutaneous tumors from each vehicle or apilimod-treated (*n* = 5 per group) mouse were combined for analysis. Data plotted are mean ± s.d. *P* value is determined by Mann-Whitney U test. **b)** MC38 tumors (mm^3)

treated with vehicle versus apilimod (*n* = 6 tumors per group) in NSG™ mice. Data plotted are mean ± s.e.m. *P* value is determined by Mann-Whitney U test on day 15 of treatment. **c)** MC38 tumors (mm^3) in mice treated with vehicle versus apilimod (*n* = 6 tumors per group) treated with IgG2a or αIL-12p40 blocking antibody. **d)** MC38 tumors (mm^3) in mice treated with vehicle versus apilimod (*n* = 6 tumors per group) treated with IgG1 or αIFNγ blocking antibody. Data for **c-d** are plotted are mean ± s.e.m. and *p* value is determined by ANOVA after post-hoc Tukey adjustment for multiple comparisons on day 15 of treatment. **e)** B16F10 tumors (mm^3) in mice treated with vehicle or apilimod (30 mg/kg daily) (*n* = 10 tumors per group, Day 9). **f)** B16F10 tumors (mm^3) with vehicle versus apilimod (*n* = 10 tumors per group, Day 11) in NSG™ mice. Data for **e-f** are mean ± s.e.m and *p* value is determined by Mann Whitney U test. MFI of **(g)** MHC-I (H2-kb, H2-kd) and **(h)** MHC-II (MHC-IA-IE) on DMSO or apilimod-treated cDCs +/- PolyI:C (50 μg/ml) or LPS (50 ng/ml) treated for 20 hours. Images are representative of two experiments. **i)** B16F10-OVA tumors (mm^3) following 21 days of pre-treatment with vehicle versus apilimod +/- subcutaneous injection of water versus PolyI:C (100μg on Day 1 and Day 14) (*n* = 8 tumors per group, assessed to Day 14 after tumor inoculation). **j)** Subcutaneous tumor volume of B16F10-OVA tumors (mm^3) with vehicle or apilimod +/- subcutaneous injection of water versus PolyI:C (100μg once weekly) (*n* = 6 tumors per group, assessed to Day 11 of treatment). Data plotted are mean ± s.e.m. *P* value is determined by Mann-Whitney U test. All *P* values are two-sided. Source data are provided in Supplementary Data 9.

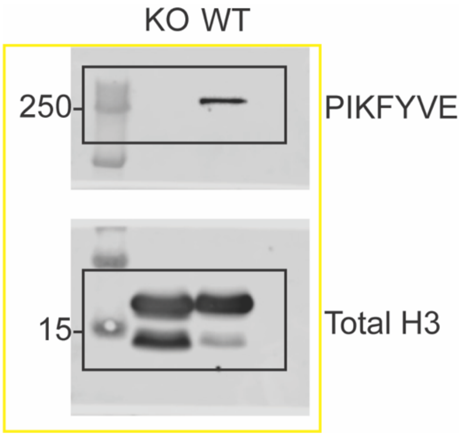

Supplementary Information 11: Source data for immunoblot in Supp 3c. Black box shows where image was cropped for the figure panel. Yellow box represents blots run on the same gel.
