# Supplementary material for: PIKfyve, expressed by CD11c-positive cells, controls tumor immunity
Source: Nat Commun. 2024 Jun 28;15:5487. doi: 10.1038/s41467-024-48931-9 (PMC11213953; doi:10.1038/s41467-024-48931-9)
Supplement: Supplementary file 3 — Description of Additional Supplementary Files [file 41467_2024_48931_MOESM3_ESM.pdf]

Description of Additional Supplementary Files

File Name: Supplementary Data 1
Description: Differentially expressed genes in PIKfyve KO versus WT cDCs

File Name: Supplementary Data 2
Description: MSigDB curated genesets ("C2") PIKfyve KO versus WT cDCs

File Name: Supplementary Data 3
Description: MSigDB Hallmark genesets ("H") PIKfyve KO versus WT cDCs

File Name: Supplementary Data 4
Description: Validated NF-κB downstream gene targets

File Name: Supplementary Data 5
Description: Differentially expressed genes in Apilimod versus DMSO-treated cDCs at 3 hours post-treatment

File Name: Supplementary Data 6
Description: Differentially expressed genes in Apilimod versus DMSO-treated cDCs at 8 hours post-treatment

File Name: Supplementary Data 7
Description: MSigDB Hallmark genesets ("H") Apilimod versus DMSO-treated cDCs at 3 hours post-treatment

File Name: Supplementary Data 8
Description: MSigDB Hallmark genesets ("H") Apilimod versus DMSO-treated cDCs at 8 hours post-treatment

File Name: Supplementary Data 9
Description: Statistical Source Data for Supplementary Figures 1-10
