# Supplementary material for: PIKfyve, expressed by CD11c-positive cells, controls tumor immunity
Source: Nat Commun. 2024 Jun 28;15:5487. doi: 10.1038/s41467-024-48931-9 (PMC11213953; doi:10.1038/s41467-024-48931-9)
Supplement: Supplementary file 13 — Reporting Summary [file 41467_2024_48931_MOESM13_ESM.pdf]

nature portfolio

Corresponding author(s): Arul M. Chinnaiyan

Last updated by author(s): Apr 19, 2024

Reporting Summary

Nature Portfolio wishes to improve the reproducibility of the work that we publish. This form provides structure for consistency and transparency in reporting. For further information on Nature Portfolio policies, see our Editorial Policies and the Editorial Policy Checklist.

Statistics

For all statistical analyses, confirm that the following items are present in the figure legend, table legend, main text, or Methods section.

n/a	Confirmed	
☐	☒	The exact sample size (*n*) for each experimental group/condition, given as a discrete number and unit of measurement
☐	☒	A statement on whether measurements were taken from distinct samples or whether the same sample was measured repeatedly
☐	☒	The statistical test(s) used AND whether they are one- or two-sided<br>*Only common tests should be described solely by name; describe more complex techniques in the Methods section.*
☒	☐	A description of all covariates tested
☐	☒	A description of any assumptions or corrections, such as tests of normality and adjustment for multiple comparisons
☐	☒	A full description of the statistical parameters including central tendency (e.g. means) or other basic estimates (e.g. regression coefficient) AND variation (e.g. standard deviation) or associated estimates of uncertainty (e.g. confidence intervals)
☐	☒	For null hypothesis testing, the test statistic (e.g. *F*, *t*, *r*) with confidence intervals, effect sizes, degrees of freedom and *P* value noted<br>*Give P values as exact values whenever suitable.*
☒	☐	For Bayesian analysis, information on the choice of priors and Markov chain Monte Carlo settings
☒	☐	For hierarchical and complex designs, identification of the appropriate level for tests and full reporting of outcomes
☐	☒	Estimates of effect sizes (e.g. Cohen's *d*, Pearson's *r*), indicating how they were calculated

Our web collection on statistics for biologists contains articles on many of the points above.

Software and code

Policy information about availability of computer code

Data collection	Flow cytometry data were acquired through LSR Fortessa (BD). Immunoblot membranes were developed using chemiluminescence and detected using Li-Cor.
Data analysis	Flow cytometry data were analyzed using FlowJo or FACS DIVA (BD Biosciences) software. Statistical analysis and data representation were performed using GraphPad Prism 9 and R using the following packages: tidyverse (1.3.1), dplyr (1.0.7), ggplot2 (3.3.5), survminer (0.4.9), survival (0.4.9), limma (3.36.0), edgeR (3.32.1), fgsea (1.16.0), and Seurat (4.1.1).

For manuscripts utilizing custom algorithms or software that are central to the research but not yet described in published literature, software must be made available to editors and reviewers. We strongly encourage code deposition in a community repository (e.g. GitHub). See the Nature Portfolio guidelines for submitting code & software for further information.

Data

Policy information about availability of data

All manuscripts must include a data availability statement. This statement should provide the following information, where applicable:

- Accession codes, unique identifiers, or web links for publicly available datasets
- A description of any restrictions on data availability
- For clinical datasets or third party data, please ensure that the statement adheres to our policy

The source data generated in this study are included in the Source Data or Supplementary Information. All materials are available from the corresponding authors

upon reasonable request. Clinical sequencing data are publicly available with raw data available upon request from dbGaP phs000673.v5.p1 [https://www.ncbi.nlm.nih.gov/projects/gap/cgi-bin/study.cgi?study_id=phs000673.v5.p1.40,43 RNA-seq data newly generated in this study for in vitro analysis have been deposited in the GEO repository at NCBI under accession codes GSE235596 [https://www.ncbi.nlm.nih.gov/geo/query/acc.cgi?acc=GSE235596] and GSE235599 [https://www.ncbi.nlm.nih.gov/geo/query/acc.cgi?acc=GSE235599]. The accession code fo the Riaz et al dataset44 is GSE91061 [https://www.ncbi.nlm.nih.gov/geo/query/acc.cgi?acc=GSE91061] and also available on github [https://github.com/riazn/bms038_analysis]. The TCGA Pan-Cancer dataset is available on the publications summary website [https://gdc.cancer.gov/about-data/publications/pancanatlas]. The ascession code for the Murakami et al dataset81 is GSE149761 [https://www.ncbi.nlm.nih.gov/geo/query/acc.cgi?acc=GSE149761]. The Qian et al dataset48 is available on the authors' website [https://lambrechtslab.sites.vib.be/en]. The Sade-Feldman et al melanoma dataset49 is available on the Human Cell Atlas website [https://www.humancellatlas.org/]. The accession code for the Chow et al endometrial cancer dataset50 is GSE212217 [https://www.ncbi.nlm.nih.gov/geo/query/acc.cgi?acc=GSE212217].

Research involving human participants, their data, or biological material

Policy information about studies with human participants or human data. See also policy information about sex, gender (identity/presentation), and sexual orientation and race, ethnicity and racism.

Reporting on sex and gender	Data on the sex of patients was included in Fig. 1 (female n=41, male n=51). The findings from these analyses are not limited or specific to one sex, and sex was not considered in the study design. Gender was not reported.
Reporting on race, ethnicity, or other socially relevant groupings	Data on the race, ethnicity or other socially relevant groupings of patients was not reported in this study. The findings from these analyses are not limited or specific to one specific social group or construct.
Population characteristics	Patients ages 25-95 years with a diagnosis of cancer who received clinical therapy with an immune checkpoint blockade agent were enrolled in continuous comprehensive clinical sequencing program at the Michigan Center for Translational Pathology (MCTP MI-Oncoseq program) were included in this study.
Recruitment	This cohort has been previous published and described. Briefly, patients who received immune checkpoint blockade therapy were recruited through the University of Michigan Hospital, Ann Arbor, MI, USA, and enrolled in the continuous MCTP MI-Oncoseq program.
Ethics oversight	As stated in previously published studies, all clinical records in this study were obtained with the approval of Institutional Review Boards, and the need for patient consent was waived following Institutional Review Board protocol review (HUM00146400, HUM00139259, HUM00163915, HUM00161860, HUM00046018).

Note that full information on the approval of the study protocol must also be provided in the manuscript.

Field-specific reporting

Please select the one below that is the best fit for your research. If you are not sure, read the appropriate sections before making your selection.

☒ Life sciences ☐ Behavioural & social sciences ☐ Ecological, evolutionary & environmental sciences

For a reference copy of the document with all sections, see nature.com/documents/nr-reporting-summary-flat.pdf

Life sciences study design

All studies must disclose on these points even when the disclosure is negative.

Sample size

No sample size calculations were performed. All in vitro experiments were performed with at least three technical replicates across two independent experiments. All in vivo experiments were performed with 3-10 mice per group to detect meaningful biological differences with good reproducibility. Group sizes were selected empirically from previously published experiments and prior knowledge (Wang et al, 2019; Yu et al, 2021; Lang et al, 2019; Wei et al, 2008). All samples sizes for various assays are listed in the Methods section or the figure legends.

References:
Wang, W. et al. CD8+ T cells regulate tumour ferroptosis during cancer immunotherapy. Nature 569, 270–274 (2019).
Yu, J. et al. Liver metastasis restrains immunotherapy efficacy via macrophage-mediated T cell elimination. Nat. Med. 27, 152–164 (2021).
Tumor
Lang et al. Radiotherapy and immunotherapy promote tumoral lipid oxidation and ferroptosis via synergistic repression of SCL7A11. Cancer Discov. 9,1673-1685 (2019)
Wei et al. Tumor-induced immune suppression of in vivo T effector cell priming is mediated by the B7-H1/PD-1 axis and TGF-beta. Cancer Res. 68, 5432-5438 (2008)

Data exclusions	Previously published human sequencing data confirmed to be from pre-treatment tumor biopsies were included in clinical correlate analyses. No data were excluded for all in vitro and in vivo experiments.
Replication	For in vitro experiments, there are at least two independent biological repeats and multiple technical repeats in each. For animal studies with drug treatment, there were at least 4 biological replicates per experiment (age and sex-matched mice purchased) performed in at least 2 independent in vivo experiments. For in vivo experiments with genetic knock out mice, at least 5 co-housed litter-mate pairs (by definition age and sex-matched) were included when each pair reached 6-8 weeks of age. In the experiments when 3 littermate pairs were used per experiment, a second independent experiment was performed and the data provided as separate experiments in the manuscript. In all instances, all attempts at replicating the experiments produced similar results. Each type of in vivo vaccine study (pretreatment, combined treatment) was performed only once as exploratory, proof of concept experiments.

Randomization

For in vitro studies, treatment groups were randomly assigned at the time freshly isolated bone marrow cells were plated and were not changed when treatment was given on the culture day indicated. These experiments were completed in replicates and independent experiments. For animal studies, mice were randomly assigned to treatment groups after tumor inoculation. The starting tumor burden in the treatment and control groups was similar before treatment.

Blinding

Experiments were not performed in a blinded manner. Blinding was not possible for in vivo studies as most of the data acquisition and analysis were performed by a single person and they needed to know the treatment groups in order to perform the study. The data were observational and findings were repeated in multiple preclinical models using both genetic and pharmacologic methods.

Reporting for specific materials, systems and methods

We require information from authors about some types of materials, experimental systems and methods used in many studies. Here, indicate whether each material, system or method listed is relevant to your study. If you are not sure if a list item applies to your research, read the appropriate section before selecting a response.

Materials & experimental systems

n/a	Involved in the study	
☐	☒	Antibodies
☐	☒	Eukaryotic cell lines
☒	☐	Palaeontology and archaeology
☐	☒	Animals and other organisms
☒	☐	Clinical data
☒	☐	Dual use research of concern
☒	☐	Plants

Methods

n/a	Involved in the study	
☒	☐	ChIP-seq
☐	☒	Flow cytometry
☒	☐	MRI-based neuroimaging

Antibodies

Antibodies used

For immunoblot analysis, the following primary antibodies were used at 1:1000 dilution unless otherwise specified: MHC-I (ThermoFisher Scientific: PA5-115363 at 1:100 dilution), MHC-IA/IE (ThermoFisher Scientific: M5/114.15.2 at 1:100 dilution), CD86 (Cell Signaling: E5W6H), CD80 (Cell Signaling: E6J6N), CD40 (Cell Signaling: E2Z7), p-NF-kB p65 Ser536 (Cell Signaling: 93H1), NF-kB p65 (Cell Signaling: D14E12), p- IkB-alpha Ser32/36 (Cell Signaling: 5A5), IkB-alpha (Cell Signaling: 9242), IkB-beta (Cell Signaling: D1T3Z), IKK-alpha (Cell Signaling: D3W6N), IKK-beta (Cell Signaling: 8943), IKK-gamma (Cell Signaling: 2685), IKK-epsilon (Cell Signaling: D61F9), p-TBK-1 (Cell Signaling: D52C2), TBK-1 (Cell Signaling: D1B4), vinculin (Sigma Aldrich: V9131 at 1:2000 dilution), and total histone 3 (Cell Signaling: 96C10). The following secondary antibodies were used: host species-matched HRP-conjugated secondary antibodies (BioRad: STAR207P, 5184-2504, STAR208P at 1:10,000 dilution).

For flow cytometry analysis, the following antibodies were used: H-2Db (ThermoFisher Scientific: 28-14-8), H-2Kb (BD Biosciences: AF6-88.5), MHC-IA/IE (BD Biosciences: M5/114.15.2), CD11c (ThermoFisher Scientific: N418), CD80 (BD Biosciences: 16-10A1), CD86 (Biolegend: GL-1), XCR1 (Biolegend: ZET), SIRP1alpha/CD172a (BD Biosciences: P84), H-2kb-SIINFEKL (BioLegend: 25-D1.16), CD90.2 (BD Biosciences: 53-2.1), CD8 (BD Biosciences: 53-6.7), CD4 (BD Biosciences: RM4.5), CD3 (BD Biosciences: 17A2), CD45 (BD Biosciences: 30-F11), CD45R (BD Biosciences: RA3-6B2), F4/80 (BD Biosciences: T45-2342), CD11b (ThermoFisher Scientific: M1/70), IFNgamma (BD Biosciences: XMG1.2), granzyme B (BD Biosciences: GB11), CD44 (BD Biosciences: IM7), CD62L (BD Biosciences: MEL-14), KLRG1 (BD Biosciences: 2F1), CD49a (BD Biosciences: Halpha31/8), TIM-1 (BD Biosciences: 5D12), PD-1/CD279 (BD Biosciences: J43), Ki67 (BD Biosciences: B56), CD69 (BD Biosciences: H1.2F3), and IL-12p40/p70 (BD Biosciences: C15.6). For tetramer staining, iTAg Tetramer/PE against H-2 Kb OVA SIINFEKL (MBL TB-5001-1) and H-2 Kb-restricted MuLV p15E KSPWFTTL (MBL TS-M507-1) were used.

Validation

All antibodies used for flow cytometry and immunoblot analyses are well-recognized clones in the field and validated by the manufacturers as follows:
Western blot antibodies:
MHC-I (ThermoFisher Scientific: PA5-115363): Validation performed by manufacturer in B16F10 cells.
MHC-IA/IE (ThermoFisher Scientific: M5/114.15.2): Validation reported by manufacturer in 6 published studies. Furthermore, validation of this clone has been performed by Fisher Scientific (a subsidiary of ThermoFisher Scientific) has been validated by western blot
CD86 (Cell Signaling: E5W6H): Validation performed by manufacturer in multiple mouse cell types and in mouse spleen.
CD80 (Cell Signaling: E6J6N): Validation performed by manufacturer in multiple mouse cell types with and without lipopolysaccharide treatment.
CD40 (Cell Signaling: E2Z7): Validation performed by manufacturer in mouse macrophage cell line with and without lipopolysaccharide treatment.
p-NF-kB p65 Ser536 (Cell Signaling: 93H1): Validation performed by manufacturer in human and mouse cell lines with and without TNF-alpha treatment.
NF-kB p65 (Cell Signaling: D14E12): Validation performed by manufacturer in human and mouse cell lines with and without TNF-alpha treatment.
p- IkB-alpha Ser32/36 (Cell Signaling: 5A5): Validation performed by manufacturer in a mouse cell line with and without TNF-alpha treatment.
IkB-alpha (Cell Signaling: 9242): Validation performed by manufacturer in a mouse cell line with and without TNF-alpha treatment.
IkB-beta (Cell Signaling: D1T3Z): Validation performed by manufacturer in multiple mouse cell lines.
IKK-alpha (Cell Signaling: D3W6N): Validation performed by manufacturer in multiple mouse cell lines.

IKK-beta (Cell Signaling: 8943): Validation performed by manufacturer in multiple mouse cell lines.
IKK-gamma (Cell Signaling: 2685): Validation performed by manufacturer in multiple human and mouse cell lines.
IKK-epsilon (Cell Signaling: D61F9): Validation performed by manufacturer in multiple mouse cell lines.
p-TBK-1 (Cell Signaling: D52C2): Validation performed by manufacturer in a human macrophage cell line with report of validation in mouse lysates in 60 published studies.
TBK-1 (Cell Signaling: D1B4): Validation performed by manufacturer in multiple mouse cell lines.
Vinculin (Sigma Aldrich: V9131 at 1:2000 dilution): Validation performed by manufacturer in multiple human and mouse cell lines and lysates.
Total histone 3 (Cell Signaling: 96C10): Validation performed by manufacturer in multiple mouse cell lines.

Flow cytometry antibodies:
H-2Db (ThermoFisher Scientific: 28-14-8): Validation performed by manufacturer in C57BL/6 mouse splenocytes.
H-2Kb (BD Biosciences: AF6-88.5): Validation performed by manufacturer in C57BL/6 mouse splenocytes.
MHC-IA/IE (BD Biosciences: M5/114.15.2): Validation performed by manufacturer in mouse splenocytes with negative expression on CD3+ cells.
CD11c (ThermoFisher Scientific: N418): Validation performed by manufacturer in mouse splenocytes with negative expression on CD45R/B220+ cells.
CD80 (BD Biosciences: 16-10A1): Validation performed by manufacturer in mouse splenocytes stimulated with lipopolysaccharide.
CD86 (Biolegend: GL-1): Validation performed by manufacturer in mouse splenocytes stimulated with lipopolysaccharide.
XCR1 (Biolegend: ZET): Validation performed by manufacturer in mouse splenocytes with dual expression of CD8alpha+.
SIRP1alpha/CD172a (BD Biosciences: P84): Validation performed by manufacturer in cultured myeloid cells from bone marrow against CD11b.
H-2kb-SIINFEKL (BioLegend: 25-D1.16): Validation performed by manufacturer in C57BL/6 mouse splenocytes pulsed with SIINFEKL.
CD90.2 (BD Biosciences: 53-2.1): Validation performed by manufacturer in mouse thymocytes.
CD8 (BD Biosciences: 53-6.7): Validation performed by manufacturer in mouse splenocytes against CD3e.
CD4 (BD Biosciences: RM4.5): Validation performed by manufacturer in mouse splenocytes against CD3e.
CD3 (BD Biosciences: 17A2): Validation performed by manufacturer in mouse splenocytes and thymocytes against CD3e.
CD45 (BD Biosciences: 30-F11): Validation performed by manufacturer mouse splenocytes.
CD45R (BD Biosciences: RA3-6B2): Validation performed by manufacturer mouse splenocytes and thymocytes against CD3e.
F4/80 (BD Biosciences: T45-2342): Validation performed by manufacturer mouse splenocytes against CD11b.
CD11b (ThermoFisher Scientific: M1/70): Validation performed by manufacturer in mouse splenocytes.
IFNgamma (BD Biosciences: XMG1.2): Validation performed by manufacturer in mouse splenocytes treated with PMA and ionomycin.
Granzyme B (BD Biosciences: GB11): Validation performed by manufacturer in mouse splenocytes treated with PMA and ionomycin.
CD44 (BD Biosciences: IM7): Validation performed by manufacturer in mouse bone marrow cells.
CD62L (BD Biosciences: MEL-14): Validation performed by manufacturer in mouse bone marrow cells and lymphocytes.
KLRG1 (BD Biosciences: 2F1): Validation performed by manufacturer in mouse splenocytes.
CD49a (BD Biosciences: Halpha31/8): Validation performed by manufacturer in C1300 mouse cell line.
TIM-1 (BD Biosciences: 5D12): Validation performed by manufacturer in mouse splenocytes.
PD-1/CD279 (BD Biosciences: J43): Validation performed by manufacturer in mouse splenocytes.
Ki67 (BD Biosciences: B56): Validation performed by manufacturer in human Jurkat and PBMCs. Reported to be tested in mouse samples.
CD69 (BD Biosciences: H1.2F3): Validation performed by manufacturer in mouse splenocytes treated with PMA.
IL-12p40/p70 (BD Biosciences: C15.6): Validation performed by manufacturer in mouse bone marrow-derived macrophages treated with interferon gamma and lipopolysaccharide.

Eukaryotic cell lines

Policy information about cell lines and Sex and Gender in Research

Cell line source(s)	MC38 cell line was acquired from Walter Storkus as previously described. B16F10 and MCA-205 were purchased from ATCC. Ovalbumin-expressing B16F10 (B16F10-OVA) and MC38 (MC38-OVA) were established with pCI-neo-mOVA plasmid (Addgene plasmid #25099) and selected with 1 mg/ml of G418 for 2 weeks as previously described.
Authentication	Cell lines were not authenticated.
Mycoplasma contamination	All cells were biweekly tested for mycoplasma contamination using the MycoAlert PLUS Mycoplasma Detection Kit (Lonza) and were found to be continually negative. More details are included in the Methods section
Commonly misidentified lines (See ICLAC register)	No cell line used in the paper is listed in ICLAC database.

Animals and other research organisms

Policy information about studies involving animals; ARRIVE guidelines recommended for reporting animal research, and Sex and Gender in Research

Laboratory animals	All mice were maintained under specific pathogen-free conditions and co-housed under standard dark/light cycles, humidity and temperature conditions which were not change throughout the study.<br><br>Female or male wild type C57BL/6J mice (Strain #: 000664), Pikfyvef/f mice (Strain #: 029331), ItgaxTg/0 (Strain #: 007567) mice, OT-I TCR transgenic mice (Strain #:003831), OT-II TCR transgenic mice (Strain #:004194), Batf3-/- mice (Strain #: 013755), and NSGTM (Strain #: 005557) were purchased from The Jackson Laboratory. ItgaxTg/0 Pikfyvef/f C57BL/6 mice were bred internally and

genotyped according to the standard protocol provided by The Jackson Laboratory.

For in vivo studies in genetic conditional knock-out models, eight- to twelve-week-old male (MC38, MC38-OVA, MCA-205) and female (MCA-205) sex-matched littermate pairs of Pikfyvef/f and ItgaxTg/0 Pikfyvef/f were used for all tumor studies.

For drug therapy studies, six- to eight -week-old male and female wild type C57BL/6 mice were inoculated with MC38 or B16F10 syngeneic cancer cell lines, respectively.

For vaccine strategy experiments, six- to eight -week-old female wild type C57BL/6 mice were inoculated with B16F10-OVA tumors.

Wild animals	None
Reporting on sex	Findings are applicable to both sexes and both sexes were used in animal studies.
Field-collected samples	None
Ethics oversight	All research in this studies were performed in compliance with all ethical regulations from the accredited nstitutional Animal Care and Use Committee at the University of Michigan, Ann Arbor who approved the study protocols.<br><br>All animal studies were conducted with the approval of the Institutional Animal Care and Use Committee at the University of Michigan prior to initiation of procedures and data collection.

Note that full information on the approval of the study protocol must also be provided in the manuscript.

Plants

Seed stocks	N/A
Novel plant genotypes	N/A
Authentication	N/A

Flow Cytometry

Plots

Confirm that:

☒ The axis labels state the marker and fluorochrome used (e.g. CD4-FITC).

☒ The axis scales are clearly visible. Include numbers along axes only for bottom left plot of group (a 'group' is an analysis of identical markers).

☒ All plots are contour plots with outliers or pseudocolor plots.

☒ A numerical value for number of cells or percentage (with statistics) is provided.

Methodology

Sample preparation	Cultured dendritic cells were collected and prepared as single cell suspensions. Single cell suspensions of mononuclear cells were isolated from bilateral tumors combined for each mouse with mechanical disassociation followed by Ficoll separation. Single cell suspensions of mononuclear cells were mechanically disassociated from bilateral tumor-draining lymph nodes.<br><br>Surface staining was performed by adding antibodies to single cell suspensions in MACS buffer (PBS, 2% FBS, 1 mM EDTA) for 30 minutes. For cytokine staining, cells were stimulated with phorbol myristate acetate (5 ng/ml), ionomycin (500 ng/ml), brefeldin A, and monensin at 37°C for 4 hours followed by surface and intracellular staining with Foxp3 /Transcription Factor Staining Buffer Set (eBioscience) per the manufacturer’s protocol. For tetramer staining, cells were first incubated with tetramer for 30 minutes prior to the addition of antibodies for surface staining.
Instrument	All data were acquired through LSR Fortessa (BD Biosciences).
Software	All data were analyzed with FlowJo or FACS DIVA (BD Biosciences) software.
Cell population abundance	Purity (>90%) of sorted or enriched cells were confirmed by flow cytometry.
Gating strategy	For splenic cells, the cells were gated on FSC-H/FSC-W and SSC-H/SSC-W based on the location known to include the cell population and to exclude doublets and then gated on CD3+CD8+ for CD8+ T cells and on CD45+ cells from which a

population of CD45R-CD3-CD90- cells for myeloid cells. For primary dendritic cells, the cells were gated on FSC-H/FSC-W and SSC-H/SSC-W based on the location known to include the cell population and to exclude doublets and then gated on CD11c+ cells. For OT-I and II cells, the cells were gated on FSC-H/FSC-W and SSC-H/SSC-W based on the location known to include the cell population and to exclude doublets and then gated on CD90+CD8+ cells. For intratumoral CD8+ T cells, the cells were gated on FSC-H/FSC-W and SSC-H/SSC-W based on the location known to include tumors and lymphoid cells, then gated on CD90+7-AAD- cells, and then CD90+CD3+ cells and then on CD8+ cells. For dendritic cells from tumor-draining lymph nodes, the cells were gated on FSC-H/FSC-W and SSC-H/SSC-W based on the location known to include tumors and myeloid cells, then gated on CD45+ cells from which a population of CD45R-CD3-CD90- cells were further gated on CD11c+ and then MHC-II+ cells.

☒ Tick this box to confirm that a figure exemplifying the gating strategy is provided in the Supplementary Information.
